# Extraction of Hard Cataract

**Published:** 1866-01

**Authors:** Gustavus Hay


### EXTRACTION OF HARD CATARACT.

By GUSTAVUS HAY, M.D.

**Read before the Boston Society for Medical Improvement, and communicated for the** *Boston Medical and Surgical Journal.*

The operation of extraction of cataract, even in cases in which the amount of success expected is sufficient to decidedly indicate the operation, presents considerable variety both as to the facility with which it is effected and as to its results. In conjunction with cataract the eye may be variously diseased and so altered that the lens may not slip out in the usual manner, and apart from the contingencies of the healing period the improvement in vision may be less than it would have been had the cataract been the only disease. We must not, however, attribute to disease in the eye what may be due to defect in the operation. Slight variations in the movement of the instruments, such as may very easily occur, become of considerable importance, owing to the minuteness of the parts, and may influence the result more or less favorably.

In illustration of the above, I have selected the following cases:—

Case I.—Mrs. E., American, æt. 57, healthy, entered the infirmary Oct. 2d, 1864. The right eye had begun to get dim three years previously; the cataract was apparently hard, and now nearly ripe. The left was noticed to be dim a year previously; its cataract also hard, but not quite ripe.

Oct. 5th.—The ordinary flap-extraction, downwards, was done on the right eye, without ether.

10th.—Patient sitting up.

18th.—Yesterday complained of slight pain. The pupil a little smaller than in the other eye. Atropine instilled.

31st.—Wound well healed. Pupil opposite the middle of the cornea, and nearly round in form. With convex 4, vision at twenty feet $\frac{1}{2\frac{1}{2}}$

Nov. 1st.—With convex $3\frac{1}{2}$, vision is $\frac{1}{2}$.

Here we have a case which, with the exception of the slight irritation recorded Oct. 18th, proceeded without any disagreeable circumstances and resulted successfully; so that a month after the operation the patient could leave the infirmary, well. But such a favorable state of things is not the rule, at least not without numerous exceptions.

Case II.—Mrs. T., American, æt. 73, entered the infirmary Oct. 6th, 1865. Sight began to fail in the left eye four years ago, with pain, which had recurred at times ever since. The right began to fail half a year after the other. Patient now sees with each eye, large objects. Each lens is somewhat opaque in parts throughout, and shows a well-defined, small nucleus. Tension of eyes not increased.

Oct. 9th.—Iridectomy was done upwards on the left eye.

23d.—Under ether, an upper section of moderate size was made at the boundary between cornea and sclerotic, in the expectation that the small nucleus would easily pass out; but, after opening the anterior capsule, the lens refused to start. The section, though slightly smaller than usual, was yet thought to be sufficiently large. Some other method than the usual one must, then, be adopted. Schuft's spoon, it was thought, would endanger, by its too great size, a rupture of the hyaloid. Critchett's vectis spoon was preferred; but owing to its flatness, it had previously occurred to me that it would bring along the lens better if modified by the addition of a small oval opening, into which the convexity of the posterior surface of the lens might settle a little. Such an opening, 2 lines long and $1\frac{1}{2}$ lines broad, had therefore been made in the middle of the bowl of the spoon, the long axis of the opening coinciding with that of the spoon. This fenestrated spoon was introduced behind the lens, and with it the greater portion of the lens brought out without losing any vitreous. It was noticeable that, notwithstanding the appearance of a well-defined, small nucleus before the operation, yet after extraction the nucleus adhered rather firmly to the cortical portion.

Nov. 1st.—Some pain last night, and redness to-day.

13th.—Pain and redness have been considerable, but now diminishing. Sight improving.

21st.—Pain nearly gone; only slight redness remaining; with convex $3\frac{1}{2}$, has vision 1-10 at ten feet.

As causes for the lens not slipping out easily, Stellwag mentions a flap too small, or a section carried too flat through the cornea, a spasmodic contraction of the sphinctor pupillæ and posterior synechiæ. He does not mention an unusual degree of adhesion between lens and capsule, which may have existed in the case just reported. As to the requisite of the section, he says:—"A section of half the circumference of the cornea is never necessary, even for large nuclear cataracts; it suffices to enter and come out somewhat under the horizontal diameter of the cornea, and to carry the knife so that the outer edge of the flap may be all along about a-quarter of a line distant from the limbus conjunctivalis. If the nucleus is small and the cortical softened, or even if the nucleus be somewhat larger, but of normal consistency, a flap is sufficient whose extent exceeds only a little a-third of the circumference of the cornea." This last is not strange if we remember that the average diameter of the lens, is four lines, and that of the cornea five lines; so that an arc of a-third of the circumference of the cornea would have a chord slightly greater than the diameter of the lens. Some additional length is generally wanted on account of the thickness of the lens.

Case III.—Mrs. T., æt. 68, American, in feeble health, entered the infirmary May 13th, 1864, with cataract of the left eye. Iridectomy was done upwards on the 14th. Patient left on the 6th of June, doing well.

Aug. 19th.—Came back. Since leaving has been ill some of the time, but better for the past six weeks. The right eye shows some lenticular opacity and small floating opacities in the viterous.

22d.—More than three months after the iridectomy, extraction was done upwards (under ether). Nucleus small.

25th.—Some pain. Lids very slightly swollen. Some conjunctival injection. Pupil dilated by yesterday's atropine. Compressive bandage continued. Atropine instilled. Weather very warm. May sit up.

27th.—Five days after operation. Conjunctiva somewhat swelled. Pupil dilated; its aperture in part of a cloudy, creamy hue. Compressive bandage omitted.

29th.—About the same, except appearance of pus in anterior chamber. Pain at times severe.

Sept. 10th.—Nineteen days from the operation. Œdema of upper lid increasing. For several days pus has shown itself near the aperture in the iris made by the iridectomy. Pupil still dilated.

27th.—Inflammation diminished. Occasional pain. Cornea slightly opaque. Pupil nearly closed. Opaque substance blocking up the artificial pupil and the natural one. Tension of globe diminished.

Oct. 2d.—Movable reddish fluid in anterior chamber. Tension much less than normal.

4th.—Patient left.

25th.—Came back. Tension restored somewhat. Reddish fluid absorbed. Cornea clear, except above in neighborhood of section. Pupil occupied by a whitish membrane.

Nov. 28th.—Iridectomy downwards and inwards.

Dec. 15th.—With convex $3\frac{1}{2}$, has vision 1-10 at ten feet.

1865, Oct. 6th.—Vision about the same, though on trial not quite as good as Dec. 15th, 1864.

This case is interesting as showing how tedious may sometimes be the inflammatory process after extraction, and also that notwithstanding very unpromising symptoms a useful amount of vision may sometimes be obtained. In consideration of the above case and of another similar one, in which the inflammatory mass occupying the pupil seemed continuous on the one hand with the edges of the artificial pupil, and on the other with the corneal section, the question occurred whether the inflammation might not be aggravated by the presence of this artificial pupil. Is it possible that this pupil, by allowing the inflamed lens-remnants to come in contact with the corneal section thereby promotes the transference of the inflammation from one to the other, or that the edges of the artificial pupil take on inflammation more readily than the rest of the surface of the iris? However, if we consider the morbid process in the two cases referred to as consisting in great part of inflammation of the portion of the lens remaining behind after extraction, it must be remembered that by means of the artificial pupil the extraction of the soft part of the lens is much facilitated. Setting this advantage of the iridectomy against the possible disadvantage referred to, there would still be reasons for considering flap-extraction preceded at an interval of some weeks by iridectomy as in general the easiest and safest method for ordinary hard cataract, inasmuch as by this method, with comparatively slight danger of rupturing the hyaloid, the removal of the lens is facilitated, and for a given amount of pressure on the eye a somewhat smaller section would suffice, and also rapid union is promoted. If the patient were quite infirm or restless, we might substitute for the usual section a smaller one of about a-third of the circumference of the cornea, and bring out the lens with the aid of the spoon, setting the advantage of the smaller section over against the danger from the use of the spoon.—*Boston Med. & Surg. Journal.*

